# Association of glomerular C4d deposition with various demographic data in IgA nephropathy patients; a preliminary study

**DOI:** 10.12860/jnp.2015.04

**Published:** 2015-01-01

**Authors:** Hamid Nasri, Ali Ahmadi, Mahmood Rafieian-kopaei, Bahman Bashardoust, Parto Nasri, Muhammed Mubarak

**Affiliations:** ^1^Department of Nephrology, Division of Nephropathology, Isfahan University of Medical Sciences, Isfahan, Iran; ^2^Department of Epidemiology, Shahid Beheshti University of Medical Sciences, Tehran, Iran; ^3^Medical Plants Research Center, Shahrekord University of Medical Sciences, Sharhekord, Iran; ^4^Department of Internal Medicine, Imam Khomeini Hospital, Ardabil University of Medical Sciences, Ardabil, Iran; ^5^School of Medicine, Isfahan University of Medical Sciences, Isfahan, Iran; ^6^Department of Histopathology, Sindh Institute of Urology and Transplantation (SIUT), Karachi, Pakistan

**Keywords:** C4d, Complement, Glomerulopathy, IgA nephropathy, Immunohistochemistry

## Abstract

*Background:* IgA nephropathy (IgAN) is the most prevalent primary chronic glomerulopathy worldwide. Thus, it is of vital importance to search for factors aggravating the disease progress, monitor disease activity and predict disease-specific therapy. C4d is a well-known biomarker of the complement cascade with a potential to meet the above needs.

*Objectives:* The aim of our study was, therefore, to determine whether C4d staining at the time of kidney biopsy had any correlation with the demographic, clinical and biochemical variables in IgAN.

*Patients and Methods:* The definition of IgAN requires the presence of diffuse and global IgA deposits which were graded ≥2+ and weak C1q deposition. C4d immunohistochemical staining was conducted retrospectively on 29 renal biopsies of patients with IgAN, which were selected randomly from all biopsies. C4d immunohistochemical staining was performed on 3-μm deparaffinized and rehydrated sections of formaldehyde-fixed, paraffin-embedded renal tissues.

*Results:* Of 29 selected patients, 68% were male. In this study, 54.2±25 percent of glomeruli in all biopsies were positive for C4d. The mean and standard deviation (SD) of serum creatinine and the magnitude of proteinuria were 1.72±1.2 mg/dl and 1582±1214 mg/day, respectively. In this study, we observed statistically significant correlations of percent C4d positivity with the serum creatinine (r=0.61, p=0.0005), magnitude of proteinuria (r=0.72, p=0.0001), the proportion of globally sclerotic glomeruli (r=0.43, p=0.02) and the proportion of tubulointerstitial fibrosis (r=0.54, p=0.0023).

*Conclusions:* The results from our investigation on C4d positivity in biopsy-proven cases of IgAN are in accord with some of the previous studies. These findings, however, require further validation in larger samples.

**Implication for health policy/practice/research/medical education:**

In a study on 29 selected IgA nephropathy patients , we found that C4d immunostaining has a significant positive correlation with serum creatinine, level of proteinuria, proportion of globally sclerotic glomeruli and quantity of tubulointerstitial fibrosis in patients with IgAN. Staining for C4d could be incorporated into the routine analysis of kidney biopsies in patients with IgAN, given its prognostic implication. Our findings, however, require further investigation with larger samples.

## 1. Background

IgA nephropathy (IgAN) is the most common glomerulopathy in the world (1,2). The diagnosis of IgAN is based on the preponderance of IgA deposits, with C3 in the mesangial area of the glomeruli (3-6). Low intensity of IgG or IgM deposits may also be found. C1q deposition is typically absent and its absence is a diagnostic clue for the immunohistological diagnosis of the disease. In some patients, the mesangial deposits may extend to capillary walls too (2-4). Since IgAN is the most prevalent primary chronic glomerulopathy and it leads to end-stage renal disease (ESRD) in up to 30-40% of cases, it is of vital importance to search for factors influencing the disease progress, monitoring the disease activity and predicting disease-specific therapy (6). The recent Oxford Classification of IgAN recognized mesangial hypercellularity, endocapillary proliferation, segmental glomerulosclerosis, and tubular atrophy/interstitial fibrosis, as independent predictors of outcome (7-10). However, other morphologic variables and immunostaining data may have significant importance and require further investigation (7-12).

While IgAN is supposed to be associated with immunoglobulin deposition in the kidney, the exact pathogenesis still remains undetermined (13,14). Additionally, immune complex-mediated complement activation via the classical pathway plays a key role in the pathogenesis of various glomerulonephritides (14-19). In summary, the classical pathway is commenced by the binding of the complement C1 complex to the antigen-antibody complex or C-reactive protein, which is followed by the cleavage of the complement component C4 into C4a and C4b, and then C4b is cleaved into C4c and C4d (18-20).

Recently much attention has been directed towards the detection and significance of C4d mesangial deposits and its association with various clinical and morphologic lesions in glomerulopathies; however, few studies have been published on this subject (18-21). C4d is also generated by activation of the lectin pathway. It has been found that patients with IgAN can be divided into two groups on the basis of the pattern of complement activation. Activation of the lectin pathway of complement is associated with more severe kidney disease. Glomerular deposition of C4d is an indicator of activation of the lectin pathway of complement (18-21). Hence, C4d is a well-known biomarker of the complement cascade. Moreover, C4d staining is an inexpensive and easy method for the analysis of kidney biopsies (18-23).

Though C4d itself has no biological function, it has established role for its indication of complement activation through the classical, alternative or lectin pathway (18-21). In one study Espinosa *et al*. proposed that C4d is a valuable tool for the differential diagnosis of membranous nephropathy (24). Thus, it is reasonable to extend the study of C4d to other immunologic diseases, including IgAN, to find the role of C4d and its clinical implication.

## 2. Objectives

The aim of our study was therefore to determine whether C4d staining at the time of the kidney biopsy had any correlation with the clinical and biochemical variables in IgAN.

## 3. Patients and Methods

### 3.1. Definition of IgAN

The pathologic diagnosis of IgAN requires demonstration of IgA-dominant or codominant mesangial or mesangial-capillary immune deposits through immunofluorescence (IF) microscopy with subdominant, or weak deposits for C_1_q. The immune deposits were semiquantified from 0 to 3+ positive intensity of brightness (23,24). The definition of IgAN requires the presence of diffuse and global IgA deposits which were graded ≥2+ and weak C_1_q deposition (23-25). All kidney biopsies, conducted at various medical centers, were sent to a reference laboratory. None of the patients was treated before the biopsy. Biopsies with less than eight glomeruli were excluded from the investigation. None of the patients was diagnosed as primary IgAN, having a history of collagen vascular diseases, diabetes or liver cirrhosis based on a questionnaire filled at the time of biopsy admission, laboratory data in patients’ records and a brief history provided by referee physicians at the time of biopsy admission.

### 3.2. Histologic data

All renal biopsies were prepared for light and direct IF microscopy. Tissue was fixed in 10% formalin for histologic sectioning. Each kidney biopsy was prepared by cutting paraffin blocks into 3-μm sections and staining for periodic acid Schiff (PAS), hematoxylin and eosin (H&E), Jones silver stain and trichrome (23-25). Each slide contained 2-3 sections. The specimen for IF was snap-frozen in liquid nitrogen. Sections (5µ in thickness) were stained for IF study with IgG, IgM, IgA, C3, and C_1_q. IF slides were reported on a scale of 0-3+ bright intensity and by a nephropathologist (23-25). IF study was performed before reviewing the slides for light microscopy unaware of patients’ data.

### 3.3. Definitions of morphologic variables

Total number of glomeruli and the number of glomeruli with global sclerosis were recorded for each biopsy. The presence of (I) mesangial hypercellularity (M), (II) endocapillary proliferation (E), (III) segmental glomerulosclerosis (S) and (IV) the proportion of tubular atrophy and interstitial fibrosis (IF/TA) was assessed as published for Oxford classification of IgAN (23-25).

### 3.4. C4d staining and histological study

C4d immunohistochemical staining was conducted retrospectively on 29 of renal biopsies of patients with IgAN, who were selected by systematic random sampling from all biopsies. C4d immunohistochemical staining was performed on 3-μm deparaffinized and rehydrated sections of formaldehyde-fixed, parafin-embedded renal tissues, using rabbit polyclonal anti-human C4d (Biomedica, Vienna, Austria) as the primary antibody, diluted 1:50 in antibody diluent (Dako, Denmark). In order to break the cross-linking of the proteins which were caused during tissue processing and to unmask the target protein epitopes, heat-induced antigen retrieval (HIER) was performed in advance using microwave (10 min at high power, 10 mM citrate buffer, pH 6.0). After cooling down for 15 min at room temperature, endogenous peroxidase was blocked with 3% H_2_O_2_ for 5 min, followed by washing the sections in TBS. Anti-C4d antibody was applied for 30 min at room temperature, followed by washing in TBS. The detection system used was Dako EnVision (Dako, Glostrup, Denmark) applied for 30 min at room temperature, followed by washing in TBS. The signal was detected with DAB chromogen (Dako, Glostrup, Denmark) for 15 min, resulting in a brown color. Following the washing step in tap water for a few min, slides were counterstained with hematoxylin (Dako, Glostrup, Denmark) for 2 min. After dehydrating, the slides were cover-slipped using mounting medium. C4d immunohistochemical staining was assessed by the percent of C4d positivity in the glomeruli in each biopsy, through evaluation of all glomeruli in every fragment. More than trace positivity of C4d in the glomeruli was considered as positive.

### 3.5. Clinical studies and laboratory data

In this retrospective histological study, the medical records of patients were reviewed to obtain various demographic, clinical and laboratory information at the time of biopsy and for follow-up activities. Data gathered at the time of biopsy included race, gender, age, serum creatinine (Cr) and proteinuria (based on a 24-h urine collection).

### 3.6. Ethical issues

1) The research followed the tenets of the Declaration of Helsinki; 2) informed consent was obtained; and 3) the research was approved by the institutional review board.

### 3.7. Statistical analysis

The mean values and standard deviations (mean ±SD) were calculated. To obtain a correlation matrix for quantitative variables in the dataset was used the Spearman’s correlation. Data were analyzed by Stata software version 12 (Stata Corporation, College Station, Texas). P values of less than 0.05 was assumed to be significant (p<0.05).

## 4. Results

**Figure 1:**
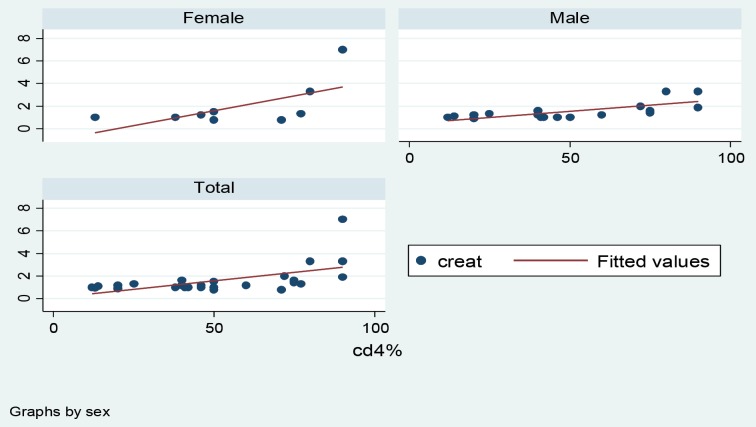
Correlation of C4d with serum creatinine

**Figure 2:**
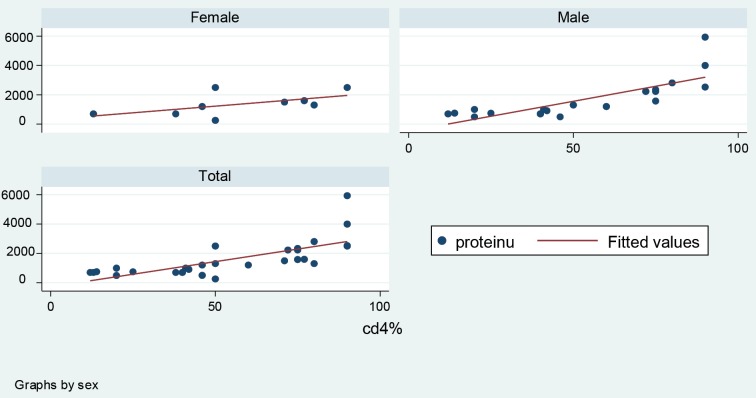
Correlation of C4d with quantity of proteinuria

**Figure 3:**
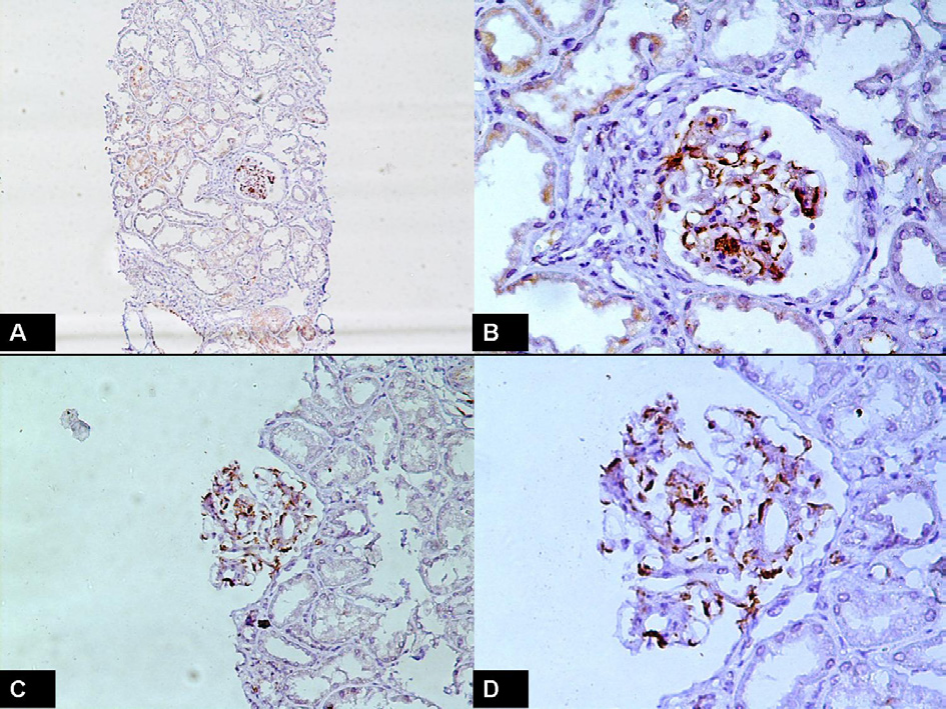
C4d immunohistochemical staining in IgA nephropathy. A) Low-power view showing glomerular positivity of C4d. The surrounding parenchyma is negative. (C4d, ×100). B) High-power view showing predominantly mesangial positivity of C4d. (C4d, ×400). C) Medium-power view showing mesangial and membranous positivity of C4d. (C4d, ×200). D) High-power view showing both mesangial and capillary wall positivity of C4d. (C4d, ×400).

Of 29 selected patients, 68% were male and 32% female. The mean±SD, minimum and maximum of age of patients were 35.03 (±11.7), 17 and 56 years. The mean ±SD of serum creatinine and quantity of proteinuria were 1.72±1.2 mg/dl and 1582±1214 mg/day, respectively. In this study, 54.2±25% of all glomeruli were positive for C4d. In this study, a significant positive correlation of C4d with serum creatinine (r=0.61, p=0.0005; Figure 1), quantity of proteinuria (r=0.72, p=0.0001; Figure 2), proportion of globally sclerotic glomeruli (r=0.43, p=0.02) and quantity of tubulointerstitial fibrosis (r=0.54, p=0.0023) was seen. The 95% confidence intervals of these are shown in Table 1. Representative photomicrographs of C4d positivity by IHC are shown in Figure 3.

**Table 1:** Correlation (r) and 95% confidence intervals (CI) between C4d and serun creatinine, level of proteinuria/day, proportion of globally sclerotic glomeruli, and quantity of tubulointerstitial fibrosis.

**Variable**	**C4d**		
	**r**	**CI 95%**	**P**
Creatinine (mg/dl)	0.61	0.31-0.79	0.0005*
Proteinuria/day	0.72	0.48-0.86	0.0001*
Proportion of globally sclerotic glomeruli (number)	0.43	0.35-0.68	0.02*
Tubulointerstitial fibrosis (%)	0.54	0.22-0.75	0.0023*

*P value less than 0.05 and was significant.

## 5. Discussion

Positive C4d staining in the glomeruli may be correlated with functional damage related to glomerular filtration and poor kidney outcome (18-22). It has been detected that patients with IgAN can be divided into two groups on the basis of the mode of complement activation. Activation of the lectin pathway of complement is correlated with more severe kidney disease (18-22). Glomerular deposition of C4d is an indicator of activation of the lectin pathway of complement (18-22). In a retrospective investigation by Espinosa *et al*., comprising of patients with IgAN who underwent kidney biopsy, the baseline age, gender, presence of macroscopic hematuria, hypertension, serum creatinine and glomerular filtration rate, urine protein, mesangial C4d staining, glomerulosclerosis, interstitial fibrosis and extracapillary proliferation were investigated (24). They found that overall 32.2% were C4d positive and 67.8% C4d negative. They detected that C4d-positive staining was univariately associated with evolution to end-stage kidney failure. Moreover, kidney survival at 10-years was 43.9% in C4d-positive patients versus 90.9% in C4d-negative patients (24). They interpreted that, negative mesangial C4d-staining in glomeruli in IgAN helps to define patients with a good long-term prognostic outcome (24). In a retrospective study including 23 IgAN patients, C4d staining and medical records including gender, age, and urine albumin were reviewed by Maeng and colleagues (25). Thirteen patients (56.5%) were positive for C4d staining in the glomerulus (25). They found that glomerular C4d deposition was associated with albuminuria (25). These findings are in accord with the results of our study too. More recently, in a retrospective investigation consisting of 283 patients with IgAN, by Espinosa *et al*., C4d was investigated (26). The study showed that 109 patients and 174 patients were classified as C4d-positive and C4d-negative, respectively. Kidney survival at 20-years was 28% in C4d-positive patients versus 85% in C4d-negative patients. They concluded that, C4d-positive staining is an independent risk factor for the development of end-stage kidney failure in IgAN patients (26).

## 6. Conclusions

In conclusion, our investigation shows that C4d immunostaining has a significant positive correlation with serum creatinine, level of proteinuria, proportion of globally sclerotic glomeruli and quantity of tubulointerstitial fibrosis in patients with IgAN. Staining for C4d could be incorporated into the routine analysis of kidney biopsies in patients with IgAN, given its prognostic implication. Our findings, however, require further investigation with larger samples.

## Authors’ contributions

All authors wrote the paper equally.

## Conflict of interests

The authors declared no competing interests.

## Funding/Support

None.
